# Relationship between self-esteem and suicidal ideation before and during COVID-19 in a non-clinical sample: mediating effects of psychological distress and hopelessness

**DOI:** 10.3389/fpsyt.2023.1240715

**Published:** 2023-09-07

**Authors:** Nguyen Tan Dat, Nobuyuki Mitsui, Satoshi Asakura, Yutaka Fujii, Kuniyoshi Toyoshima, Ichiro Kusumi

**Affiliations:** ^1^Department of Psychiatry, Hokkaido University Graduate School of Medicine, Sapporo, Japan; ^2^Health Care Center of Hokkaido University, Sapporo, Japan

**Keywords:** COVID-19, self-esteem, hopelessness, psychological distress, suicidal ideation

## Abstract

**Introduction:**

Several studies have highlighted the impact of the novel coronavirus disease (COVID-19) pandemic on suicide. Accordingly, investigating the risk factors of suicide during this crisis is important. Based on the escape theory of suicide, the current study examined the serial mediating roles of psychological distress and hopelessness in the relationship between self-esteem and suicidal ideation. It also aimed to explore whether or not the COVID-19 pandemic changed the mediation effect in any way.

**Methods:**

Data were collected from 645 university students before and during the pandemic. The study employed mediation and multi-group analyses to test the hypotheses.

**Results:**

The results demonstrated that individuals with low self-esteem reported high psychological distress, which further lead to hopelessness and eventually heightened suicidal ideation. Multi-group analysis revealed that psychological distress exerted a greater impact on suicidal ideation during COVID-19.

**Discussion:**

The finding suggested that self-esteem, hopelessness, and psychological distress could help elucidate the development of suicidal ideation. Clinicians may target these factors in suicide prevention programs, particularly in the settings of the COVID-19 pandemic or future crisis.

## 1. Introduction

The current coronavirus disease (COVID-19) pandemic has led to a surge in mental health issues worldwide (1). Mental health conditions, such as anxiety, depression, or even posttraumatic stress disorder, do not only affect patients with COVID-19 but also emerge from the general population (2). Moreover, the psychological aftereffects of the pandemic are likely to last for months or perhaps years (3). One of the most serious mental issues that have increased due to COVID-19 is suicidal behaviors. Compared with the pre-COVID-19 era, significant increases in incident rates for suicidal thoughts (10.81%) and suicide attempts (4.68%) were observed during the pandemic (4). Furthermore, a moderate prevalence of suicidal ideation among university students was also reported in the midst of COVID-19 (5, 6). In Japan, despite experiencing a downward trend before the pandemic (2011–2018), the suicide rates of students continuously increased during the pandemic period (7). These results suggested that additional studies on suicide among university students during COVID-19 should be conducted.

The link between COVID-19 and increased suicide risk is a result of complex societal and psychological changes due to the pandemic. Research demonstrates that the negative effects of COVID-19, such as financial instability, feelings of uncertainty, or reduced social support, would create pandemic-related stress. Psychological responses to pandemic-related stress would then lead to an increase in suicidal ideation (8).

The escape theory of suicide (9) provides a framework for understanding the development of suicidal ideation, especially during the current pandemic. According to the theory, an individual must undergo a six-step process for suicide to emerge. (a) First, they must fail to meet standards due to high expectations or recent setbacks or both. (b) In the next step, the individual then attempts to use self-blame strategies to make sense of perceived failures. (c) An aversive state of self-awareness emerges in the third step due to these self-blaming behaviors. (d) Negative emotions, especially depression, and anxiety, also begin to surface following the increase in negative self-awareness. (e) The person then responds to these unfavorable emotions by attempting to escape from meaningful thoughts into a numb state of cognitive deconstruction. (f) Finally, suicidal ideation manifests as an extension of the desire of the person to escape from the self.

The reason why we believe the escape theory of suicide is applicable to the settings of the COVID-19 outbreak is because COVID-19 has created significant changes and setbacks for countries around the world, which is similar to the conditions mentioned in the first step of the theory. Specifically, the COVID-19 pandemic has led to unfavorable societal changes, such as inflicting damage to local economies, instilling fear of contagion in the minds of the people and creating disruptions in daily relationships (8), which have been linked to an increase in suicidal ideation. According to the first step of escape theory, recent or current adverse conditions that indicate a significant decline from earlier conditions would lead to suicide. As specified by Baumeister (9), examples of adverse conditions are economic downturn, failing health, or deterioration of relationships, which are consistent with those of Hwang et al. (8) on societal changes during the COVID-19 crisis that were linked with suicide. Consequently, the current study believes that the COVID-19 crisis has created substantial setbacks for every country and its population, which caused an increase in suicidal behaviors.

The second and third steps of the theory emphasized how self-blame and negative self-awareness characterize suicidal individuals. These steps could be represented by low self-esteem. Particularly, low self-esteem was linked to a high tendency toward self-blaming behaviors (10, 11). In addition, studies reported that individuals with low self-esteem were significantly more self-conscious about negative emotions than those with high self-esteem (12). Regarding the relationship between self-esteem and suicidality, scholars suggested that low self-esteem is a prominent risk factor for all suicidal behaviors (13). The results of a cross-national comparison study (14) indicated that countries with people with relatively low levels of self-esteem are more likely to have higher suicide rates regardless of other social factors. Furthermore, enhancing self-esteem, especially among adolescents and young adults, is considered an effective strategy for reducing suicidal behaviors (15, 16). Accordingly, to create an effective self-esteem intervention program for reducing suicidal behaviors, additional studies on the role of self-esteem and its underlying mechanism in the development of suicide are highly needed. The current study hypothesized that self-esteem is a predictor of suicidal ideation.

The fourth stage of the theory proposes that negative emotions, emerge due to negative self-awareness. Due to previous stages of the theory, the individual now views themselves as inadequate, and thus negative feelings, especially anxiety and depression, arise (9). This stage could be measured by psychological distress, which is an emotional state characterized by anxiety and depression symptoms (17). Additionally, high levels of psychological distress indicate a potential diagnosis of depressive and anxiety disorders (17). Psychological distress is also associated with an increased risk of suicide, particularly in the midst of the COVID-19 pandemic (18, 19). Finally, previous studies suggested that the concept of cognitive destruction in the fifth step is characterized by a feeling of hopelessness. Specifically, individuals with high hopelessness are indicated to only focused on the present, short-term oriented, and have a concrete focus on immediate actions, which is similar to the state of cognitive deconstruction (20, 21). Hopelessness is represented by the assumption that bad things will happen and that good things will not happen together with the conviction that nothing can be done to improve the situation (22). With regard to the relationship between hopelessness and suicide, prior studies indicate that hopelessness is a strong predictor of suicidal behaviors, even in Japanese sample (23, 24). Hopelessness is also believed to be a better predictor of suicide behaviors than depression, and thus is an important component of the fifth step of escape theory (20). Hence, the current study examines psychological distress and hopelessness as serial mediators in the relationship between self-esteem and suicidal ideation.

Based on the abovementioned literature review, the present study aimed to explore the relationship among self-esteem, hopelessness, psychological distress, and suicidal ideation. Furthermore, within the framework of the escape theory of suicide, we investigated a serial mediation model with self-esteem as the independent variable, hopelessness and psychological distress as the serial mediators, and suicidal ideation as the dependent variable. We hypothesized that psychological distress and hopelessness would serially mediate the relationship between self-esteem and suicide. Finally, we further explored whether or not a significant difference exists between the pre-pandemic and the pandemic mediation model.

## 2. Methods

### 2.1. Participants

The initial sample consisted of data from 932 participants who were students that visited the Hokkaido University Health Care Center, Japan before and throughout the COVID-19 pandemic. Specifically, data were collected during the academic year (from April to March), from 536 students before the pandemic (2018–2019) and from 396 students during the pandemic (2020–2021). Data was collected from students who had visited the university health care center during this period to receive psychiatric consultation. The current study only utilized non-clinical data, because scholars suggest that the presence of any mental disorder is a major risk factor for suicide (25), which makes determining the relationship between the study variables difficult. Furthermore, one study reported that data collected from the Hokkaido University Health Care Center may lead to bias toward a clinical sample (26). Thus, to ensure the non-clinical status of the current sample, the study conducted an initial screening evaluation using the Mini International Neuropsychiatric Interview (MINI) Screen (version 5) and a clinician’s diagnosis to exclude participants suspected of having major psychiatric disorders (27). The MINI Screen is a self-rated scale that consists of 16 modules, in which each refers to a diagnosis of a major psychiatric disorder (28). As suicidal ideation is the dependent variable, the current study did not use the suicide module. After excluding data by comparing the results of the MINI Screen and the diagnosis of the clinician, the final sample comprised data from 645 participants, among which 291 (45.1%) were collected during the pandemic. The participants were all Japanese, and no missing data points were observed.

### 2.2. Ethical considerations

Data were collected as part of routine medical examinations, such that consent forms were not obtained. However, the participants were given the option to decline participation by opting out of having their information used. The opt-out process was modified in accordance with the steps listed on the website of the Hokkaido University Health Care Center (information is presented in Japanese). No students apply to have their data opt out from the study. The ethical committee of the Graduate School of Medicine in Hokkaido University approved the study, which was conducted according to the ethical guidelines outlined by the Helsinki Declaration in 1964 (amended in Fortaleza on October 2013).

### 2.3. Measures

#### 2.3.1. Self-esteem

The study used the Rosenberg Self-Esteem Scale (RSES) to measure the self-esteem of the participants (29). The RSES consists of 10 items (e.g., I feel that I have a number of good qualities), which are rated using a four-point Likert-type scale. The study used the Japanese version of the scale, which was adapted by Mimura and Griffiths (30). For the current sample, the data demonstrated very good reliability (Cronbach’s alpha = 0.85).

#### 2.3.2. Psychological distress

The Kessler Psychological Distress Scale (K10) was used to assess psychological distress (31). The K10 is a 10-item scale (e.g., During the last 30 days, about how often did you feel worthless?) that assesses non-specific anxiety and depressive symptoms of the past 4 weeks. The Japanese version of the scale exhibited good reliability and validity (32). In the current study, the internal consistency of the scale was 0.89, which indicates good reliability.

#### 2.3.3. Hopelessness

The study employed the Beck Hopelessness Scale (BHS) to assess feelings of hopelessness (33). The BHS is a 20-item scale that assesses future hopelessness using true or false statements such as “I do not expect to get what I really want.” The Japanese adaptation of the scale by Tanaka et al. (34) was used, which displayed a high internal consistency for the current sample (0.84).

#### 2.3.4. Suicidal ideation

Suicidal ideation was assessed using the ninth item of the Patient Health Questionnaire (PHQ-9), which asks about “thoughts that you would be better off dead or hurting yourself in some way” (35). Scholars suggested that the ninth item is effective in screening for suicidal ideation or suicide attempt (36, 37). The Japanese adaptation of the item was used (38), and the PHQ-9 displayed an acceptable Cronbach’s alpha (0.83) for the present sample.

### 2.4. Data analysis

First, the study used the measures of skewness and kurtosis to check for normality of data with skewness between [−3, 3] and kurtosis between [−10, 10], which were considered acceptable (39). To determine whether or not a significant difference exists between the groups of participants tested before and during the COVID-19 outbreak, the study used chi-square and *t*-test. Two-tailed Pearson’s correlation was then performed to explore relationships between the study variables. Data were analyzed using JMP Pro 16.

Path analysis was conducted in AMOS 25 to test the mediation model. In the model, self-esteem was the independent variable, psychological distress and hopelessness were the serial mediator variables, and suicidal ideation was the dependent variable. Standardized path coefficients (β) were reported. The model was just identified (0 degree of freedom), such that model fit could not be accessed. Thus, the study followed the recommendation of MacKinnon (40) to examine the mediation effect, which requires meeting three conditions, namely, a significant association between the independent and dependent variables, a significant association between the mediators and the dependent variable while controlling for the independent variable, and a significant coefficient for the indirect path between the independent and dependent variables via the mediators. The significance of the direct and indirect effects was tested using a bias-corrected bootstrap procedure with 2,000 bootstrap samples. The direct and indirect effects can be considered significant if the 95% bootstrap confidence intervals (CI) do not include zero (39).

Finally, to determine if the mediation effects are different before and during the pandemic, the study conducted multi-group analysis using AMOS. First, the study estimated an unconstrained model (i.e., a model in which the structural parameter coefficients were freely estimated across the pre-pandemic and pandemic groups) and a constrained model (i.e., a model in which the structural parameters are equally constrained across the pre-pandemic and pandemic groups). The chi-square difference test was used to assess differences between groups in which a significant *p*-value would indicate statistically significant differences between the pre-pandemic and pandemic models. The critical ratios of the differences in parameters were further conducted using AMOS to determine which specific paths in the models are significantly different. If *z*-values are larger than 1.96, then the pre-pandemic and pandemic mediation models are suggested to significantly differ in that path. Additionally, the bootstrapping analysis with 2,000 bootstrap samples was used to determine the significance of the indirect effects of each model (41).

## 3. Results

### 3.1. Preliminary analyses

Table 1 reports the demographic information of the respondents. No variable surpassed the skewness and kurtosis values of ±2, which indicates that data were normally distributed (39). Between-group comparisons demonstrated that no significant differences exist between data collected before and during the pandemic in terms of age, self-esteem, and hopelessness. However, the pre-pandemic group displayed significantly lower levels of psychological distress and suicidal ideation than did the pandemic group. Additionally, the gender ratio between the pre-pandemic and pandemic groups was significantly different.

**Table 1:** Characteristics of the study participants.

	Total (*N* = 645)	Pre-pandemic (*N* = 354)	Pandemic (*N* = 291)	*t-*test. chi-square test	*p*-value
Gender (male/female)	414/231	242/112	172/119	5.95	0.015*
Age (mean/SD)	21.78 (2.72)	21.83 (2.80)	21.73 (2.62)	0.49	0.622
RSES (mean/SD)	21.87 (5.01)	22.03 (0.27)	21.68 (0.29)	0.88	0.377
K10 (mean/SD)	15.49 (8.73)	14.75 (0.46)	16.38 (0.51)	−2.35	0.018*
BHS (mean/SD)	11.55 (4.20)	11.46 (0.22)	11.67 (0.25)	−0.61	0.536
PHQ-9 Item 9 (mean/SD)	0.69 (0.93)	0.60 (0.83)	0.80 (1.03)	−2.65	0.008**

RSES = self-esteem; K10 = psychological distress; BHS = hopelessness; PHQ-9 Item 9 = suicidal ideation.

*p < 0.05, **p < 0.01.

Correlation analysis (Table 2) illustrated that age and gender were not significantly correlated with the other study variables. As expected, self-esteem was significantly and negatively correlated with hopelessness, psychological distress, and suicidal ideation. Additionally, the results revealed that psychological distress had a significant positive correlation with hopelessness as well as suicidal ideation. Finally, hopelessness also exhibited a significant and positive correlation with suicidal ideation.

**Table 2:** Correlation between variables.

Variables	Gender	Age	RSES	K10	BHS
**Gender**					
Age	0.04				
RSES	0.06	0.05			
K10	−0.05	−0.07	−0.53***		
BHS	0.02	−0.07	−0.65***	0.47***	
PHQ-9 Item 9	−0.07	−0.08	−0.39***	0.47***	0.39***

RSES = self-esteem; K10 = psychological distress; BHS = hopelessness; PHQ-9 Item 9 = suicidal ideation.

***p < 0.001.

### 3.2. Mediation analysis

In a model that includes only the direct path from self-esteem to suicidal ideation, self-esteem significantly predicted suicidal ideation (β = −0.39, 95% CI: −0.44 to −0.34, *p* < 0.001). This model explained 16% of variance in suicidal ideation.

**Figure 1:**
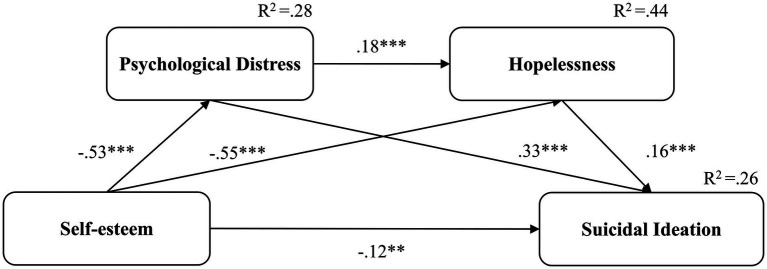
Serial mediation model for the full sample with standardized coefficients. **p < 0.01, ***p < 0.001.

In a model containing the two mediators, the results illustrated that self-esteem continue to be significantly and negatively associated with suicidal ideation (β = −0.12, 95% CI: −0.21 to −0.03, *p* = 0.008). In addition, psychological distress and hopelessness were significantly and positively related with suicidal ideation (β = 0.33, 95% CI: −0.24 to −0.40, *p* < 0.001; β = 0.16, 95% CI: 0.07 to 0.25, *p* = 0.002, respectively). Finally, the paths from self-esteem to psychological distress (β = −0.53, 95% CI: −0.58 to −0.46, *p* < 0.001), from self-esteem to hopelessness (β = −0.55, 95% CI: −0.61 to −0.48, *p* < 0.001), and from psychological distress to hopelessness (β = 0.18, 95% CI: −0.10 to 0.25, *p* < 0.001) were significant (Figure 1). The total indirect effect of self-esteem on suicidal ideation (Table 3) was significant (β = −0.27, 95% CI: −0.35 to −0.21, *p* < 0.001). The model explained 26% of variance in suicidal ideation. Accordingly, the serial mediation model was supported.

**Table 3:** Bootstrapping results for the standardized direct and indirect effects.

Model pathways	Point estimates	95% CI		*p*-value
		Lower	Upper	
**Full sample model**				
**Direct effects**				
RSES → PHQ-9 Item 9	−0.12	−0.21	−0.03	0.008
RSES → BHS	−0.55	−0.61	−0.48	0.001
RSES → K10	−0.53	−0.58	−0.46	0.001
K10 → BHS	0.18	0.10	0.25	0.001
K10 → PHQ-9 Item 9	0.33	0.24	0.40	0.001
BHS → PHQ-9 Item 9	0.16	0.07	0.25	0.002
Total indirect effect from RSES → PHQ-9 Item 9	−0.27	−0.35	−0.21	0.001
**Pre-pandemic model**				
**Direct effects**				
RSES → PHQ-9 Item 9	−0.18	−0.30	−0.04	0.009
RSES → BHS	−0.54	−0.63	−0.45	0.001
RSES → K10	−0.53	−0.60	−0.44	0.001
K10 → BHS	0.17	0.08	0.27	0.002
K10 → PHQ-9 Item 9	0.25	0.13	0.36	0.001
BHS → PHQ-9 Item 9	0.09	−0.03	0.22	0.158
Total indirect effect from RSES → PHQ-9 Item 9	−0.19	−0.28	−0.10	0.001
**Pandemic model**				
**Direct effects**				
RSES → PHQ-9 Item 9	−0.07	−0.20	0.07	0.322
RSES → BHS	−0.57	−0.65	−0.48	0.001
RSES → K10	−0.52	−0.59	−0.42	0.002
K10 → BHS	0.18	0.08	0.28	0.001
K10 → PHQ-9 Item 9	0.39	0.28	0.49	0.001
BHS → PHQ-9 Item 9	0.23	0.10	0.35	0.001
Total indirect effect from RSES → PHQ-9 Item 9	−0.35	−0.45	−0.26	0.001

RSES = self-esteem; K10 = psychological distress; BHS = hopelessness; PHQ-9 Item 9 = suicidal ideation.

### 3.3. Multi-group analysis

To examine whether the serial mediating effect of psychological distress and hopelessness in the relationship between self-esteem and suicidal ideation is consistent between the pre-pandemic and pandemic groups, the study conducted multi-group analysis. The results from chi-square difference test demonstrated that the differences between the constrained and unconstrained models were significant [χ^2^ diff (6) = 16.09, *p* = 0.01], which indicates that statistically significant differences exist between the pre-pandemic and pandemic models.

**Figure 2:**
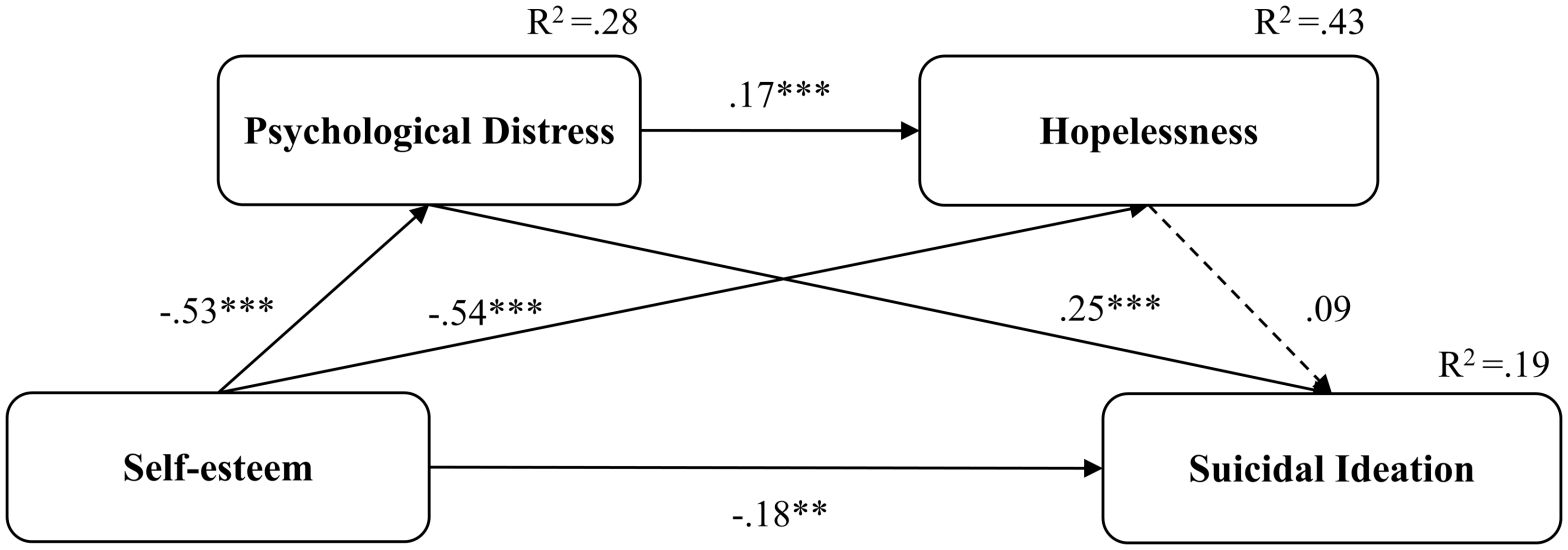
The pre-pandemic serial mediation model with standardized coefficients. **p < 0.01, ***p < 0.001. Dotted lines represent non-significant coefficients; solid lines represent significant coefficients.

**Figure 3:**
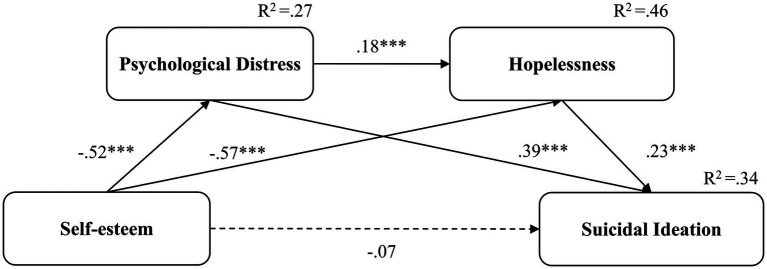
The pandemic serial mediation model with standardized coefficients. ***p < 0.001. Dotted lines represent non-significant coefficients; solid lines represent significant coefficients.

In the pre-pandemic model, the model (Figure 2) explained 19% of variance in suicidal ideation. The study observed a significant direct effect (β = −0.18, 95% CI: −0.30 to −0.04, *p* = 0.009) and a significant total indirect effect (β = −0.19, 95% CI: −0.28 to −0.10, *p* < 0.001) of self-esteem on suicidal ideation. The pandemic model (Figure 3) explained 34% of variance in suicidal ideation. The study noted a non-significant direct effect (β = −0.07, 95% CI: −0.20 to 0.07, *p* = 0.322) and a significant total indirect effect (β = −0.35, 95% CI: −0.45 to −0.26, *p* < 0.001) of self-esteem on suicidal ideation. Table 3 presents the specific direct and total indirect effects for all models. Additionally, the non-significant direct effect of the pandemic model indicated that psychological distress and hopelessness fully mediated the relationship between self-esteem and suicidal ideation during the pandemic.

The critical ratios of differences in parameters were further examined to determine which pathway coefficients were significantly different between the two models (41). The results demonstrated that the pathway from psychological distress to suicidal ideation was significantly weaker for the pre-pandemic sample (*Z* = −2.374, β = 0.25, *p* < 0.001) than that for the pandemic group (β = 0.39, *p* < 0.001).

## 4. Discussion

The current study investigated the relationship among self-esteem, psychological distress, hopelessness, and suicidal ideation. Specifically, it aimed to explore the serial mediation effect of psychological distress and hopelessness on the relationship between self-esteem and suicidal ideation. Furthermore, we examined whether or not the mediation effect was significantly different between data collected before and during the pandemic.

In line with previous studies (42, 43), the current findings depicted a significant increase in psychological distress during the COVID-19 pandemic. Furthermore, the results indicated that suicidal ideation was greatly increased during this crisis. Previous scholars observed that compared to before the pandemic, suicidal ideation underwent a sharp increase in the midst of the pandemic in many countries (44–46). Apart from suicidal ideation, suicide death, suicide attempt, and self-harm, also significantly changed during COVID-19 (47, 48). Notably, however, the current result is in contrast with that of a previous study in Japan (49), which illustrated that university students experienced significantly low levels of suicidal ideation during than before COVID-19. This difference could be due to the difference in the target samples between the two studies. Ewing et al. (50) have suggested that university students without major mental health problems could experience more difficulty when addressing COVID-19-related stress and negative emotions than do students with mental health problems. Accordingly, the difference in suicidal ideation could be due to the focus of the current study, which is only on a non-clinical sample, while Otsuka et al. (49) focused on students who visited the school counseling service (which may be biased more toward a clinical sample).

Regarding the relationship between self-esteem and suicidal ideation, the current study found that self-esteem is directly and negatively associated with suicidal ideation, which is consistent with the results of previous studies (51, 52). The relationship between self-esteem and suicidal behaviors has been well established and examined in many suicide theories. For example, the interpersonal theory of suicide states that low self-esteem is related to perceived burdensomeness, which, together with thwarted belonginess, could lead to the emergence of a suicidal desire (53). Furthermore, stage three of escape theory posits that recent low self-esteem due to failure or setback may lead to suicide. Escape theory also differentiates between suicidal and non-suicidal individuals with low self-esteem. It mentions that although non-suicidal people tend to hold critical views of themselves and others, suicidal people tend to hold favorable views of others while maintaining critical perceptions of the self (9).

Moreover, the results of path analysis revealed that psychological distress and hopelessness serially mediated the associations between self-esteem and suicidal ideation. The result suggested that individuals with low self-esteem experienced high psychological distress, which further lead to feelings of hopelessness and, eventually, to the development of suicidal ideation. Separate studies demonstrated that hopelessness (54) and psychological distress (55) were mediators between self-esteem and suicidal ideation. Additionally, Wang et al. (56) conducted a study on medical students in China and reported that hopelessness and psychological distress serially mediated the relationship between psychological strains and suicidal behaviors. However, to the best of our knowledge, this study is the first to investigate the serial mediation roles of psychological distress and hopelessness in the relationship between self-esteem and suicidal ideation. The current results could be explained based on escape theory. According to the theory, failure and setback would lead to negative self-attribution and self-awareness, which are characterized by low self-esteem in the current model. A person with negative self-views would then develop negative emotions and try to escape into a numb state of cognitive deconstruction. In the mediation model of the present study, these stages were represented by psychological distress and hopelessness. Finally, this sequential chain of events would drive an individual to commit suicide (9).

The result of multi-group analysis pointed to a significant difference between the pre-pandemic and pandemic mediation models, which indicates that the serial mediation effect is stronger for data collected during than before the pandemic. This finding is consistent with the first step of escape theory, which posits that significant recent or current setbacks, such as economic instability, health threat, and disturbance in close relationships, would precede suicide (9). The public frequently experienced such events during the COVID-19 pandemic (8). Thus, an interpretation of the result is that the presence of the pandemic could strengthen the pathways of the mediation model, and living through the COVID-19 pandemic may increase the risk of suicide.

Unexpectedly, the results indicate that the link between psychological distress and suicidal ideation in the mediation model was significantly weaker pre-pandemic in comparison with data collected during the pandemic, which warrants further investigation. A possible explanation is that stressors related to COVID-19, such as self-isolation, lack of social support, and economic uncertainty (57), have strengthened the relationship between the two variables. Previous studies reported that pandemic-related stress could lead to a significant increase in depression, anxiety, and stress (58, 59). Similarly, an increase in suicide death was linked to the fear of COVID-19 (60, 61). The results of the current study also revealed that psychological distress and suicidal ideation for the pandemic group were significantly higher than those of the pre-pandemic group, which partially supports this explanation. Furthermore, a recent study suggested that non-clinical university students may experience more difficulty in addressing COVID-19-related stress and negative emotions than students with mental health problems (50). The present study conducted analysis using a cohort of non-clinical university students; thus, a possibility existed that the current sample was more affected by stress and negative emotions related to the pandemic, which led to the stronger relationship between psychological distress and suicidal ideation. Hence, the emergence of pandemic-related stressors and the use of data from a non-clinical sample may be the explanation for the strengthened link between psychological distress and suicidal ideation in the pandemic group compared with the pre-pandemic sample. However, additional research is required to put forward a more concrete explanation regarding this difference.

### 4.1. Limitations

The current study has several limitations that need to be addressed. First, the cross-sectional design makes determining the causation of factors challenging. Thus, future studies should implement a longitudinal design to precisely determine the link among self-esteem, psychological distress, hopelessness, and suicidal ideation. Second, the study used self-reported measures for obtaining data for the study variables, which could lead to respondent or information bias. Thus, we recommend that prospective studies should collect data using a structured interview design. Another limitation is related to the use of the ninth item of the PHQ-9 to measure suicidal ideation, which may raise certain concerns regarding the reliability of the results. Nevertheless, two aspects are worth mentioning: currently, the scales for measuring suicidal ideation or suicidal behaviors (e.g., the Columbia Suicide Severity Rating Scale or the Beck Scale for Suicide Ideation) has yet been validated or adapted to Japanese versions. Second, previous studies suggested that the ninth item of the PHQ-9 is effective in screening for suicidal ideation (36, 37). Finally, there was a significant difference in gender distribution between the pre-pandemic and the pandemic sample, which could influence the results.

### 4.2. Clinical implications

Although self-esteem enhancement has been suggested to reduce suicidal behaviors (15, 16), the underlying mechanism of the change in this relationship remains unclear. Study further suggests that self-esteem interventions would be more successful when its contents are theory- and evidence-based and cater to the unique requirements of different target groups (15). Thus, to create an effective self-esteem intervention program for reducing suicidal ideation, studies on the mediators and mechanisms of the change in the relationship between self-esteem and suicidal ideation are much needed.

Furthermore, self-esteem interventions have also shown promising results among university students. Recent studies suggest that self-esteem-based interventions could be effective in improving self-acceptance (62), academic achievement (63), or reducing eating disorder behaviors (64) in samples of undergraduate students. These studies target self-esteem by various methods, ranging from teaching cognitive behavior therapy (CBT) or mindfulness techniques, to psychoeducation about topic such as self-understanding or body images (62–64). However, as far as we know, currently there is no study investigate the effect of self-esteem-intervention in reducing suicidal behaviors among university students. The current results indicate that hopelessness and psychological distress are the underlying mechanism through which self-esteem affects suicidal ideation. One proposed method for reducing psychological distress and hopelessness in order to improve self-esteem is through CBT. Indeed, CBT programs that specifically develop to target low self-esteem have also show to reduce anxiety and depression, which are two factors that closely related to psychological distress and hopelessness (65).

Moreover, the results demonstrated that the relationship between variables were significantly different between data from before and during the pandemic, especially in the path from psychological distress to suicidal ideation. This result indicates that implementing a self-esteem intervention program that targets psychological distress and hopelessness is highly needed during the current COVID-19 crisis. In addition, this result may not only be limited to the present pandemic period but may also be relevant to future crisis scenarios. As previously mentioned, the first step of the escape theory of suicide states that major life events precede suicide behaviors, which can lead to fall short of expectations (9). As a result, the present study could also serve as reference in providing an explanation for the increase in suicide rates in future crisis situations.

Finally, while the current study tried to control for the influence of mental disorders by limiting the sample to only those without any mental disorders, other psychosocial factors such as personality traits or temperament, which was not evaluated in this study, could also affect the outcomes of suicidal behaviors (66, 67). Accordingly, future studies should control for the presence of these factors when examining the impact of self-esteem, psychological distress, and hopeless on suicidal behaviors.

## 5. Conclusion

In summary, this is the first study investigating the role of the escape theory in the pandemic as well as comparing the differences between the levels of self-esteem, psychological distress, hopelessness and suicidal ideation during the pre-pandemic and pandemic period. The present study found that low self-esteem is a risk factor for suicidal ideation, and psychological distress as well as hopelessness serially mediated this relationship. In addition, it found a significant difference between the pre-pandemic and pandemic models, specifically in the path from psychological distress to suicidal ideation. These findings highlight the importance of targeting self-esteem, psychological distress, and hopelessness in treating individuals with suicidal ideation, especially during the ongoing pandemic or in future similar crises.

## Data availability statement

The raw data supporting the conclusions of this article will be made available by the authors, without undue reservation.

## Author contributions

NM: conceptualization. NM and NT: study design, literature searches, and visualization. NT: data analysis and writing—original draft. NT, NM, SA, YF, KT, and IK: writing—review and editing. NM, SA, YF, KT, and IK: supervision and final script validation. All authors contributed to the article and approved the submitted version.

## Funding

This study was supported by Japan Society for Promotion of Science (JSPS) KAKENHI (to SA) (Grant Number: JP 18K0758308).

## Conflict of interest

The authors declare that the research was conducted in the absence of any commercial or financial relationships that could be construed as a potential conflict of interest.

## Publisher’s note

All claims expressed in this article are solely those of the authors and do not necessarily represent those of their affiliated organizations, or those of the publisher, the editors and the reviewers. Any product that may be evaluated in this article, or claim that may be made by its manufacturer, is not guaranteed or endorsed by the publisher.
